# A Video-Based Measurement Framework for Chewing-Event Detection Using 3D Facial Landmark Dynamics and sEMG-Based Reference Annotation

**DOI:** 10.3390/s26113351

**Published:** 2026-05-25

**Authors:** Nicola Giulietti, Carlotta Massotti, Hermes Giberti

**Affiliations:** 1 Dipartimento di Ingegneria Industriale e dell’Informazione, Università degli Studi di Pavia, Via Adolfo Ferrata 5, 27100 Pavia, Italy; hermes.giberti@unipv.it; 2 Department of Mechanical Engineering, Politecnico di Milano, Via La Masa 1, 20156 Milan, Italy; carlotta.massotti@polimi.it

**Keywords:** chewing event detection, video-based measurement system, 3D facial landmarks, surface electromyography, real-time monitoring, masticatory activity, Monte-Carlo uncertainty evaluation

## Abstract

Accurate measurement of chewing events in natural eating conditions is important for unobtrusive monitoring of feeding behavior and masticatory function. Yet, existing methods often rely on contact sensors, dedicated wearables, or manual annotation. This work presents a non-contact, video-based framework for chewing-event detection using frontal facial video, normalized 3D facial landmark dynamics, and recurrent temporal modeling. To obtain physiologically grounded reference labels, synchronized bilateral anterior temporalis surface electromyography was acquired during real-meal sessions and used to derive chewing-event annotations during dataset construction, whereas inference relied exclusively on video. Facial motion was represented from frame-wise 3D landmarks and processed by recurrent neural networks, with model selection performed through Bayesian hyperparameter optimization. On an independent hold-out test set comprising five sessions and 18,836 frames, the proposed method detected 577 chewing events versus 589 ground truth events, corresponding to a mean absolute error of 4.4 chews/session and a mean absolute percentage error of 4.32%. A comparison with a related rule-based video method from the literature showed substantially larger counting errors (MAE = 39.4, MAPE = 30.39%), particularly in sessions that included concurrent activities such as speaking, suggesting that the proposed approach can reduce counting errors relative to the considered rule-based baseline under the specific meal conditions tested in this feasibility study. The effect of landmark-localization uncertainty on the predicted chewing probability was assessed through Monte Carlo propagation, showing limited impact for most prediction instants and greater sensitivity for intermediate probability values. Finally, the ONNX implementation achieved a mean latency of 8.96 ± 5.74 ms on CPU and 6.89 ± 3.58 ms with CUDA execution on the test workstation, supporting real-time applicability. To support practical deployment, the pipeline was also implemented as a native Kotlin Android application and tested on a commercial tablet, achieving real-time operation at 20 fps.

## 1. Introduction

Chewing-event detection is a key step toward objective monitoring of both eating behavior and masticatory function in everyday life. Beyond oral-function assessment, chewing-related variables such as chew count and eating rate are associated with clinically and behaviorally relevant outcomes, including food intake, subjective appetite, and satiety-related physiological responses. These links motivate tools capable of measuring chewing dynamics outside controlled laboratory protocols [1]. At the same time, mastication assessment is increasingly recognized as relevant to healthy aging and general well-being, yet many established methods remain cumbersome, time-consuming, or difficult to scale for routine and continuous use [2]. From a sensing perspective, the central challenge is to obtain reliable and fine-grained chewing measurements while minimizing user burden and preserving deployability in natural settings. Existing approaches span contact-based biosignals, wearable sensors, acoustic methods, and vision-based techniques, but many still suffer from obtrusive sensing requirements, environmental sensitivity, or limited robustness to non-chewing mouth and jaw movements [3,4]. As a result, further methodological development and validation are still needed before robust chewing-event detection can be achieved across heterogeneous real-world settings.

To address this gap, an end-to-end pipeline for chewing-event detection is presented, based on frontal video, geometric features extracted from facial landmarks, and temporal modeling. The method is intended as a step toward operation under realistic meal conditions, namely while subjects are eating in everyday environments, and with potential suitability for deployment on common consumer devices such as laptops, tablets, and smartphones. The proposed framework is developed and evaluated through physiologically based label generation from synchronized sEMG, event-level testing on independent meal recordings, analysis of the effect of landmark-localization uncertainty, and real-time deployment on a native mobile application. For each frame, a 3D face mesh is estimated using MediaPipe, yielding a temporal sequence of facial landmarks that captures mouth and jaw motion over time [5]. These dynamics are then modeled with a recurrent neural network that outputs the probability of an ongoing chewing event at each time step. The final architecture is selected through hyperparameter optimization.

Facial landmarks are adopted instead of full video frames because, once normalized, they provide a more compact representation with greater potential for generalization. Compared with frame-based approaches, landmark-based representations are generally less sensitive to subject appearance, illumination changes, and background variability, while reducing the dependence on raw image content and supporting effective learning from limited data. Reliable ground truth annotation of chewing events from video remains difficult. Manual labeling is inherently error-prone and may be unreliable when chewing occurs with the mouth fully closed. To obtain reference annotations with a physiological basis, sEMG signals are acquired from the anterior temporalis muscle and synchronized with the video. The synchronized muscle activity is then used to derive the training labels. Since sEMG is required only during training for label generation, the proposed system operates from video alone at inference time while benefiting from supervision derived from masticatory muscle activity during training.

The model performance was evaluated on a test set and compared with the results obtained with the rule-based approach described in [6] on the same set of data. The effect of landmark-localization uncertainty on the predicted chewing probability is assessed through Monte Carlo uncertainty propagation. A native mobile implementation of the proposed approach is also presented to evaluate real-time on-device performance in a practical monitoring scenario. The resulting model should be interpreted primarily as a proof of feasibility of the proposed approach, rather than as a definitive characterization of performance under broader operating conditions, although the present results indicate that encouraging performance can already be obtained from a limited number of acquisitions.

## 2. Related Works

Non-vision-based approaches remain central in the chewing monitoring literature because they can capture mastication through signals that are physiologically or mechanically close to jaw muscle activity. Surface EMG has been used both to characterize chewing patterns in detail and to detect chewing and swallowing events in wearable systems, confirming the strong observability of masticatory activity but also highlighting practical constraints related to electrodes, skin attachment, placement, and day-to-day usability [7,8]. Closely related wearable designs attempt to reduce this burden by embedding sensing into objects already worn near the head, such as smart-glasses, earables, or eyeglass frames instrumented with microphones, IMUs, pressure sensors, or proximity-based measurements of temporalis motion [9,10,11,12]. These systems show that chewing can be detected or counted from head-adjacent wearables, but the available studies also point to recurring deployment issues: audio methods remain sensitive to microphone placement and surrounding sounds, wearable pipelines may still rely on custom hardware or task-specific form factors, and several studies report limitations in daily-life validation, limited participant numbers, imperfect labels, or unresolved robustness against simultaneous activities such as speaking, head turning, and other non-chewing motions. Even when the sensing principle is effective, the need to wear and maintain dedicated hardware can remain a barrier to unobtrusive, scalable monitoring in natural settings. These limitations motivate the growing interest in vision-based approaches, which can reduce sensor burden at inference time and exploit facial and upper-body motion without direct skin contact [4]. Within this line, some studies formulate the problem at the level of bite detection or broader eating-behavior analysis rather than directly targeting chewing-event detection. Rule-based systems based on 468 3D facial keypoints have been proposed to count bites from meal videos, with the explicit goal of reducing the burden of manual annotation, while broader autonomous vision systems have combined pose estimation and temporal action localization to estimate mouthful count, chewing duration, and hand-to-mouth behavior in home-like settings. These studies support the feasibility of camera-based monitoring, but also show that much of the literature remains focused on behavioral summaries or manually annotated video data, with the associated burden and subjectivity acknowledged by the authors themselves [13,14]. For the more specific problem of chewing analysis from facial motion, recent vision-based studies have shown that chewing-related dynamics can be extracted from video using jaw trajectories derived from MediaPipe landmarks or brightness variations in the mouth–chin region captured by a smartphone camera. However, these approaches are generally based on simplified temporal descriptors, peak-detection strategies, or waveform classification, which may limit their robustness when chewing must be distinguished from visually similar mouth movements in less constrained settings [6,15]. Existing vision-based studies suggest that chewing-related activity can be inferred from facial motion, but they also highlight recurring limitations in annotation strategy, robustness to confounding mouth movements, and reliance on handcrafted or reduced visual descriptors rather than richer temporal representations [6,15,16]. These limitations become particularly relevant when the objective is unobtrusive monitoring in realistic settings, rather than analysis in instrumented or tightly constrained conditions [13,14]. Recent video-event detection studies have increasingly explored attention-based and transformer-based architectures for modelling complex spatio-temporal patterns. For instance, Khan et al. proposed ViolenceNet, a multi-scale transformer framework with joint feature understanding for violence detection in video [17]. However, the adoption of such architectures may be less straightforward when only limited datasets are available and when real-time operation on consumer devices is required. Overall, the available literature still leaves open the need for a practical framework for chewing-event detection that combines video-only inference with physiologically based supervision and evaluation on recordings acquired during actual meal consumption. The present study addresses this need by integrating normalized 3D facial landmarks, recurrent temporal modeling, and sEMG-derived reference labels within a single deployable pipeline, developed and assessed on recordings collected during real lunch sessions and further examined through landmark-uncertainty analysis and native mobile implementation. In the proposed formulation, chewing detection is addressed as a temporal event-detection problem from frontal facial video, using facial-motion information derived from landmark sequences together with supervision having a physiological basis. Compared with raw image-based approaches, normalized facial landmarks provide a more compact and structured representation, which may reduce sensitivity to inter-subject appearance variability and support learning from a limited number of recording sessions. In contrast to approaches based on rule-based chew counting from simple geometric signals derived from a limited set of facial landmarks, the proposed framework learns chewing dynamics directly from temporal landmark patterns. It therefore avoids wearable sensors at inference time, reduces reliance on purely manual video annotation, and does not depend on handcrafted decision rules or simplified visual descriptors. Potentially confounding activities such as speaking are also considered, with the aim of improving robustness under realistic operating conditions. The computational burden remains compatible with real-time operation on commercial mobile devices.

## 3. Materials and Methods

**Figure 1:**
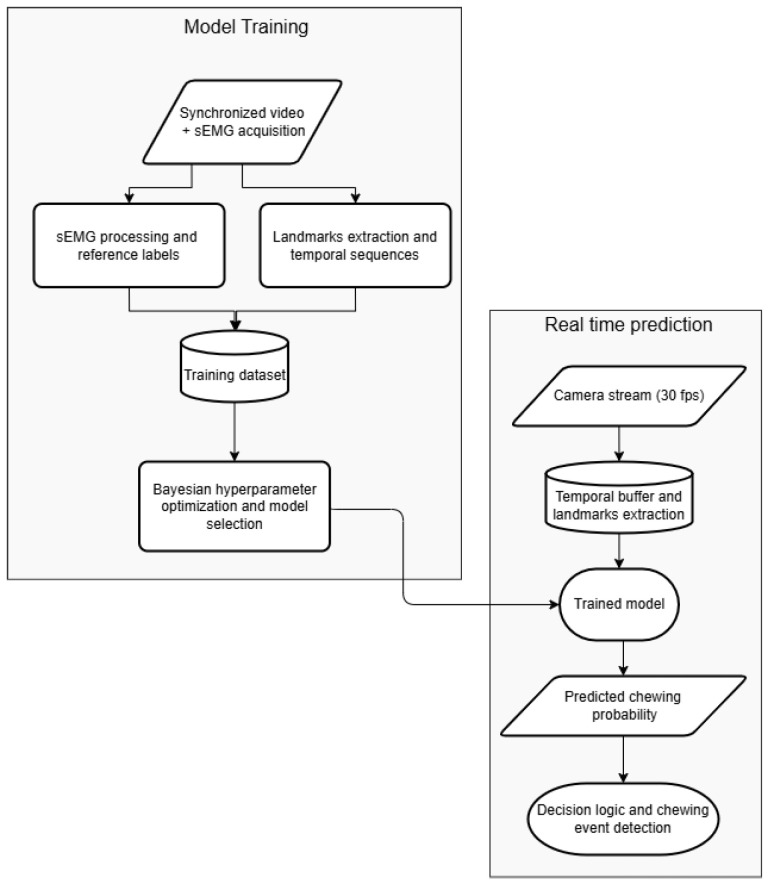
Overview of the proposed workflow, including the offline training stage and the real-time chewing detection pipeline.

This section presents the materials and the experimental and computational methods adopted for data acquisition, annotation, model development, and performance evaluation. Figure 1 summarizes the overall workflow of the proposed method, which comprises an offline training stage based on synchronized video and sEMG signals, and a real-time stage for chewing-event detection from image data.

### 3.1. Data Acquisition and Ground Truth Annotation

**Figure 2:**
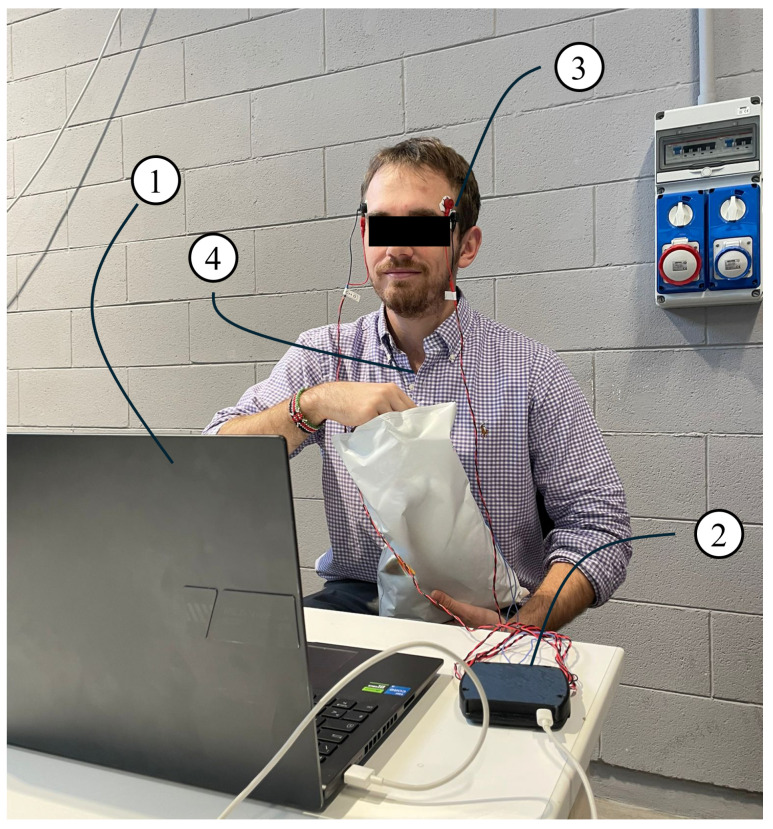
Schematic representation of the experimental setup. (1) Laptop used for data acquisition, (2) sEMG acquisition system connected to the laptop, (3) surface EMG electrodes positioned bilaterally over the anterior temporalis region, and (4) reference electrode placed over the clavicular region.

**Figure 3:**
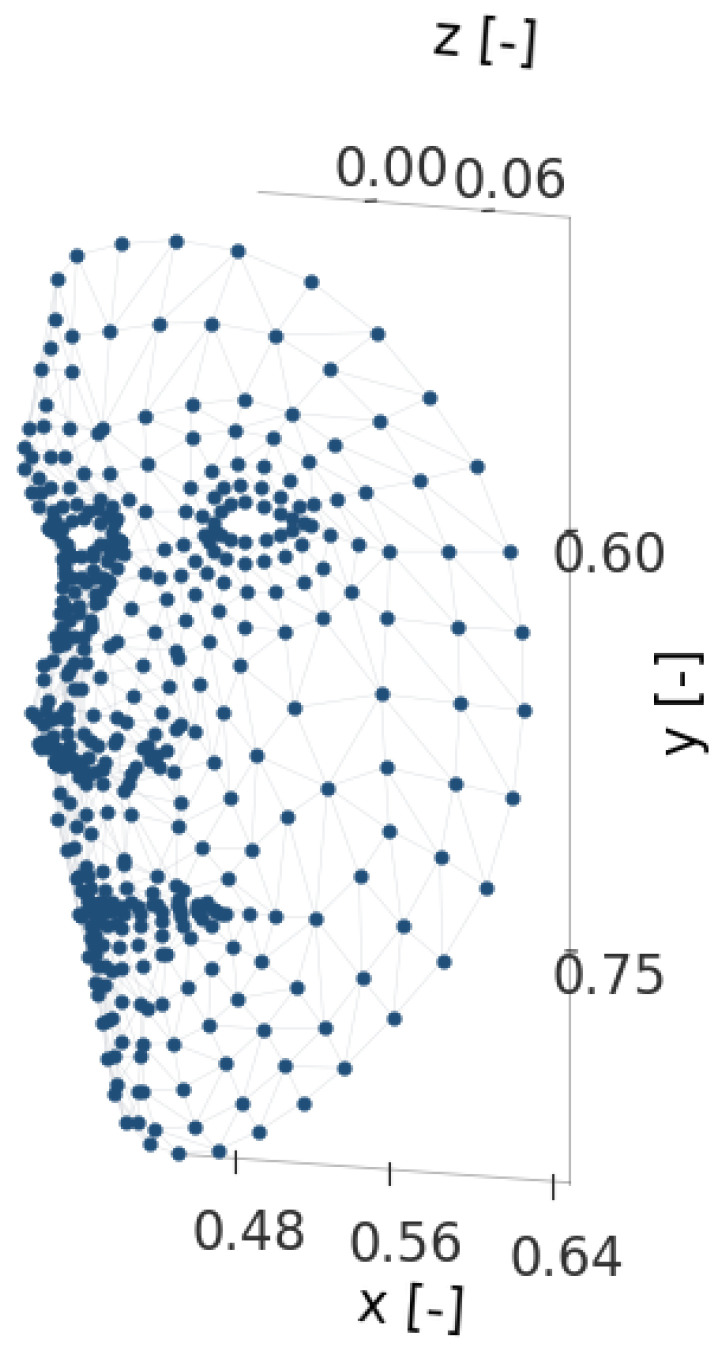
Example of the facial landmarks extracted from a frontal video frame using MediaPipe Face Mesh.

As illustrated in Figure 2, the experimental setup consisted of a laptop for synchronized data acquisition, a custom sEMG acquisition system connected to the laptop, two pairs of surface electrodes placed bilaterally over the anterior temporalis muscles, and two reference electrodes positioned over the clavicular regions. Frontal video and bilateral anterior temporalis sEMG were acquired simultaneously during the same recording session using a laptop-based setup. Video was recorded with the integrated camera of a Lenovo ThinkPad T16 Gen 2 (Lenovo, Beijing, China) equipped with a Realtek camera (Realtek Semiconductor Corp., Hsinchu, Taiwan) at 640×480 pixels and 30 fps. This relatively low spatial resolution was intentionally adopted to enable a real-time operation on standard commercial hardware with minimal latency. EMG signals were acquired at 1000 Hz using two of the eight available channels of a custom eight-channel surface sEMG acquisition board (LWT^3^ S.r.l., Milan, Italy), configured in a double-differential layout. Each channel employed three electrodes: two positioned over the target muscle and one used as reference. The analog front-end was based on the Texas Instruments ADS1298 (Texas Instruments Incorporated, Dallas, TX, USA), a 24-bit analog-to-digital converter providing integrated amplification and simultaneous multichannel digitization. Electrode placement over the anterior temporalis followed the anatomical criteria described in [18]. In particular, placement was defined from the intersection of reference lines identified using the mandibular angle, condylar head, auricle, and canthus, in order to ensure repeatable positioning across sessions. A custom acquisition software recorded video and sEMG and assigned timestamps to both streams using a shared internal clock. This enabled offline temporal synchronization between the muscle-activation signals and the corresponding video frames. For each video frame, facial motion was represented through a 3D face mesh estimated with MediaPipe Face Mesh, which provided 468 facial landmarks [19,20]. An example of the extracted landmarks is shown in Figure 3. The algorithm was configured to track a single face per frame, with both detection and tracking confidence thresholds set to 0.5. If no face was detected in a frame, the corresponding landmark coordinates were stored as missing values so that the temporal structure of the sequence could be preserved.

Ground truth chewing annotations were derived from the two temporalis sEMG channels using a semi-automatic activity-detection procedure. The automatic component was designed to accelerate and standardize the initial identification of candidate chewing-related activations, while the final reference labels were obtained after visual verification of the sEMG signals. Each EMG channel was processed independently. First, the raw EMG trace was band-pass filtered between 10 and 500 Hz. The filtered signal was then full-wave rectified, and a smooth envelope was obtained by low-pass filtering the rectified signal at 5 Hz. Following the baseline-dependent thresholding strategy adopted in [7], the decision threshold was defined from baseline statistics rather than fixed a priori. In our implementation, the baseline was taken as the first 0.5 s of the envelope for each acquisition and channel, since no chewing events occurred within this initial time window in any acquisition. The threshold was therefore set as $T=\mu_{0}+3\sigma_{0}$, where $\mu_{0}$ and $\sigma_{0}$ denote the mean and standard deviation of the baseline segment, respectively. Candidate activation intervals were identified as contiguous envelope segments above threshold. To suppress short suprathreshold fluctuations unlikely to correspond to meaningful chewing-related muscle activation, only intervals lasting at least 100 ms were retained. This choice is consistent with the temporal scale of chewing-related muscular activity reported in [21], where the occlusal/contact phase is described as being on the order of 100–200 ms within a chewing cycle lasting approximately 0.6–1 s. To limit false detections caused by channel-specific noise or transient artifacts, an automatically detected activation interval was accepted only when detected in both EMG channels. After automatic detection, all candidate annotations were visually reviewed on the band-pass filtered sEMG signals. In these signals, chewing-related temporalis activations were generally clearly distinguishable from baseline activity, facilitating visual verification of their timing in most recordings. The automatic threshold-based procedure, applied to the sEMG envelope, was therefore used to support and speed up the annotation process, rather than to replace visual verification. During the review step, thresholding errors or session-specific artifacts were corrected when necessary. The synchronized video recordings were used only as an additional cross-check in occasional ambiguous cases, for example, when the sEMG pattern required contextual confirmation. The resulting annotations, therefore, represented temporally consistent bilateral muscle activations and were used as reference labels for the synchronized video data.

**Figure 4:**
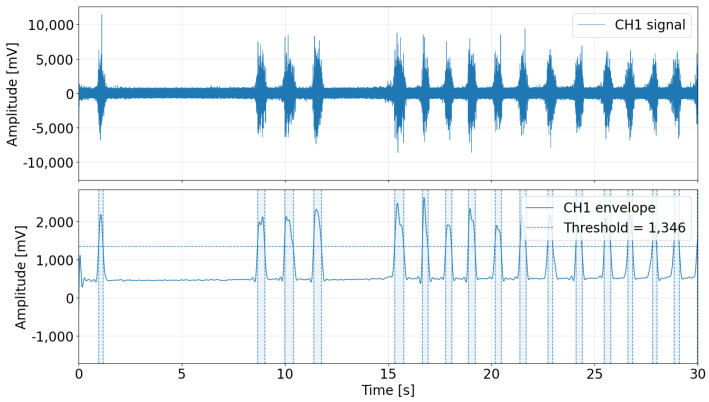
Example of annotation processing in a segment containing chewing activity. The upper subplot shows the raw anterior temporalis sEMG signal, while the lower subplot shows the corresponding envelope. The dashed horizontal line indicates the baseline-derived threshold computed as $\mu_{0}+3\sigma_{0}$ from the first 0.5 s of the envelope. Shaded regions denote detected activation intervals lasting at least 100 ms.

**Figure 5:**
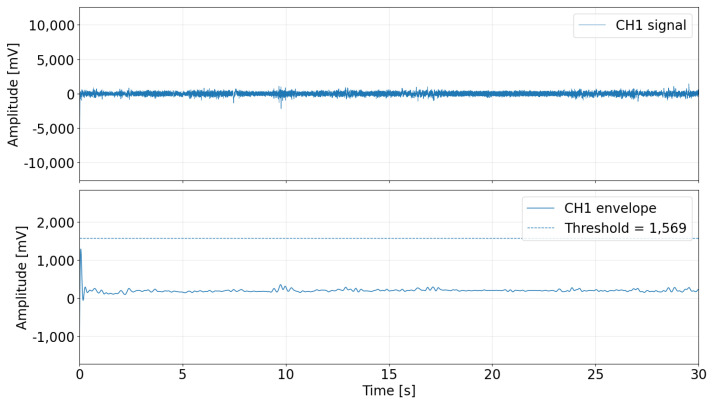
Example of annotation processing in a segment with no valid chewing activations. The upper subplot shows the raw anterior temporalis sEMG signal, while the lower subplot shows the corresponding envelope. The dashed horizontal line indicates the baseline-derived threshold computed as $\mu_{0}+3\sigma_{0}$ from the first 0.5 s of the envelope. No suprathreshold intervals satisfying the minimum duration criterion were retained.

Examples of the resulting annotation procedure are shown in Figure 4 and Figure 5. Figure 4 shows a segment containing chewing activity, whereas Figure 5 reports a segment in which no valid chewing activations were identified but the subject was speaking. In both figures, the upper subplot reports the raw sEMG trace and the lower subplot reports the corresponding envelope, with the decision threshold displayed only on the envelope plot.

### 3.2. Model Training

The collected dataset consisted of multiple synchronized acquisitions recorded across different days and eating situations, involving five subjects and different food types. A key characteristic of the dataset is that recordings were acquired under realistic conditions. Each session was collected during the participant’s regular lunch break, either at home or in the workplace, while allowing natural behavior such as chewing, speaking, grasping objects, and bringing food to the mouth, including in the presence of bulky foods such as pork ribs. Consequently, the dataset reflects everyday meal consumption conditions more closely than controlled laboratory settings, in which the environment is regulated, chewing may be temporally constrained, and food is typically standardized. Overall, the training dataset included 108,013 frames, distributed in 21 separate acquisitions. Each frame was represented by 468 MediaPipe facial landmarks, each described by three spatial coordinates, yielding a 1404-dimensional feature vector. The associated target was a binary label, where 1 indicated a chewing frame and 0 a non-chewing frame. Positive samples accounted for 25.9% of the dataset, whereas 74.1% belonged to the negative class, indicating a moderate class imbalance. In addition, 1225 frames, i.e., 1.13%, contained missing landmark values. For temporal modeling, the landmark coordinates of each frame were arranged into a one-dimensional feature vector, denoted as $x_{t}\in R^{1404}$, where *t* indicates the frame index. Missing or invalid values were replaced with zeros. Causal sliding windows of length *W* were generated so that the model input at time *t* was defined as $X_{t}=\left[x_{t-W+1},x_{t-W+2},\ldots ,x_{t}\right]$ with associated target label $y_{t}\in \left\{0,1\right\}$. In this way, the prediction at time *t* was based only on the current and preceding frames. Each acquisition was processed separately, and temporal windows were generated only within the same recording, so that no sample crossed acquisition boundaries. Each temporal window was normalized independently by centering and scaling each feature using statistics computed within that same window. For each feature, the window-specific mean was subtracted and the result was divided by the corresponding window-specific standard deviation. This window-wise normalization reduced the influence of absolute landmark position and scale, while emphasizing local temporal variations in facial motion. The adopted model was based on recurrent neural networks, selected for their capability to account for temporal correlations in sequential data acquired over consecutive frames [22]. Two recurrent formulations were considered, namely Long Short-Term Memory (LSTM) [23] and Gated Recurrent Unit (GRU) [24] networks. The model input was defined as a temporal sequence of landmark vectors, while the output was obtained from the last hidden state of the sequence through a fully connected layer providing the estimated probability of chewing at frame *t*. The final model configuration, including recurrent unit type, number of layers, and related hyperparameters, was determined by means of an automated Bayesian optimization procedure [25,26]. The hyperparameter search space included the following parameters:

- **Window size**: from 30 to 100 frames, corresponding to 1–3.33 s observation window;
- **Stride**: 1, 2, 5, 10, 20, or no overlap;
- **Recurrent layer type**: LSTM or GRU;
- **Number of recurrent layers**: from 1 to 8;
- **Hidden size**: from 32 to 512 units, with step 4;
- **Dropout rate**: from 0.0 to 0.3;
- **Learning rate**: from $10^{-6}$ to $5\times 10^{-3}$ on a logarithmic scale;
- **Batch size**: from 32 to 128, with step 4.

The model architecture was intentionally kept compact in order to limit the computational burden and preserve real-time operability on commercially available hardware. Hyperparameter optimization was carried out for up to 104 iterations. For each candidate configuration, all admissible temporal windows were generated and retained together according to the acquisition from which they originated. The dataset was then partitioned at the acquisition level, after random shuffling, into training and validation subsets with an 80/20 ratio. In this way, all windows from a given recording were assigned to only one subset, thereby preventing data leakage. The validation set was used exclusively for model selection, while the final evaluation of generalization performance was performed only on the independent test acquisitions. To assess the possible split-dependence of the selected configuration, an exploratory leave-one-subject-out validation was also performed. In each fold, all acquisitions from one subject were withheld from the training data and used for subject-wise validation, while the model was trained on the acquisitions from the remaining subjects. The recurrent architecture and hyperparameters were kept fixed to those of the selected Bayesian optimization trial, namely trial no. 102, and the Bayesian optimization was not repeated within each fold. This analysis was intended to evaluate the robustness of the selected configuration across subject-wise partitions, rather than to provide a fully nested estimate of model-selection stability. Model training was performed using the Adam optimizer [27]. To mitigate class imbalance, a larger weight was assigned to positive samples in the loss function, according to the general principle of cost-sensitive learning for imbalanced classification [28]. Let $n_{\mathrm{neg}}$ and $n_{\mathrm{pos}}$ denote the numbers of negative and positive samples in the training subset, respectively. The positive-class weight $w_{\mathrm{pos}}$ was computed according to Equation (1), so as to increase the contribution of the minority class while avoiding excessively large updates.

(1) $$w_{\mathrm{pos}}=\min\left(4.0,\;\max\left(1.2,\;\sqrt{\frac{n_{\mathrm{neg}}}{n_{\mathrm{pos}}}}\right)\right).$$

Model optimization was based on the weighted mean squared error (Weighted MSE) loss reported in Equation (2), where ${\hat{y}}_{i}$ denotes the predicted probability, $y_{i}$ the corresponding ground truth label, and $w_{i}$ the sample weight, set equal to $w_{\mathrm{pos}}$ for positive samples and to 1 for negative samples. In the binary setting, the adopted Weighted MSE corresponds to a weighted Brier-type score on the predicted probabilities [29]. This formulation allows optimization to be carried out directly on continuous probability estimates rather than on thresholded binary decisions, while leaving the final decision threshold to a subsequent event-level calibration step [30].

(2) $$L=\frac{1}{N}\sum_{i=1}^{N}w_{i}{\left({\hat{y}}_{i}-y_{i}\right)}^{2}.$$

Model training and hyperparameter optimization were carried out on a workstation equipped with an Intel Core i9-14900KF CPU (Intel Corporation, Santa Clara, CA, USA), an NVIDIA GeForce RTX 4090 GPU with 24564 MiB of VRAM (NVIDIA Corporation, Santa Clara, CA, USA), and 32 GiB of RAM, running Ubuntu 22.04.5 LTS. The software environment was based on Python 3.13.11 and PyTorch 2.10.0+cu128. Each trial was exported and stored in ONNX format [31] to preserve an inference-ready model independent of the PyTorch training runtime, thereby enabling consistent cross-trial comparison, reproducible benchmarking, and straightforward cross-platform deployment across desktop, server, and mobile environments. At inference time, the model outputs, at each frame, a sigmoid probability in the range [0, 1], representing the likelihood of an ongoing chewing event. Each estimate is computed from the current frame together with the preceding frames contained in the selected temporal window. The procedure is therefore causal and compatible with real-time operation. The final chewing decision was obtained by post-processing the probability sequence. First, each frame was classified as positive when the predicted probability exceeded a predefined threshold. Consecutive positive frames were then merged into contiguous temporal segments. A segment was accepted as a chewing event only if its duration was not shorter than a prescribed minimum length; shorter segments were rejected as spurious activations. The final decision, therefore, depended on both probability level and temporal persistence. The post-processing rule was calibrated on the validation data by means of a grid search. The probability threshold was varied from 0.50 to 0.95 with a step of 0.01, while the minimum event length was varied from 3 to 30 frames. Each parameter combination was evaluated at the event level against the ground truth annotations. Predicted and reference binary sequences were converted into chewing events and matched using a one-to-one criterion. A predicted event was considered correctly detected if it overlapped with a reference chewing event by at least one frame. The optimal configuration was selected by maximizing the event-level F1 score, consistently with the event-detection objective of the proposed method.

### 3.3. Model Performance Evaluation

The trained model was evaluated on an independent hold-out test set comprising 5 sessions, corresponding to a total of 18,836 frames. These sessions were kept acquisition-disjoint from the training and validation data, so that no recording contributed samples to more than one subset. However, the hold-out evaluation was not subject-disjoint, since the test sessions involved subjects who were also represented in the model-development data. Test samples were organized into temporal windows using the same preprocessing pipeline adopted during training (Section 3.2). Missing or non-finite landmark values were replaced with zeros before inference. For each temporal window, the model produced the estimated probability of an ongoing chewing event at the current frame. The frame-wise probability sequence was then converted into candidate chewing events using the post-processing rule calibrated on the validation dataset. Frames with probability above the selected threshold were identified, consecutive positive frames were merged into contiguous temporal segments, and segments shorter than the prescribed minimum duration were discarded. In parallel, the reference binary annotations were converted into ground truth chewing events, each defined as a contiguous segment of positive frames. A predicted event was considered correctly detected when it overlapped with a reference event by at least one video frame. Unmatched predicted and reference events were counted as false positives and false negatives, respectively. Precision, recall, and F1 score were then computed from the resulting event-level counts. For a direct comparison with a related video-based chew-counting method from the literature, an additional benchmark was carried out on the same hold-out test sessions using an implementation of the approach proposed by Kim et al. [6]. In that method, facial landmarks are extracted with MediaPipe, and a one-dimensional chewing-motion signal is computed as the average Euclidean distance between one nose landmark (i.e., landmark index 4) and five mandibular landmarks (i.e., landmark indices 176, 148, 152, 377, and 400) over time. The resulting signal is smoothed with a Savitzky–Golay filter, and chew counts are obtained by detecting local maxima using the derivative-sign rule described in the original paper. A candidate peak at frame *i* is accepted when the proportion of non-negative derivative samples within the preceding *k*-frame interval is at least 0.6, and the proportion of non-positive derivative samples within the following *k*-frame interval is also at least 0.6. In addition, the upward and downward phases are each constrained to last at least 0.2 s, and the next peak is searched only after 0.4 s in order to avoid multiple detections within the same chewing cycle. The proposed method and the literature baseline were therefore compared on the same hold-out test sessions by using the same sEMG-derived reference counts. For each test session, the predicted chew count was compared with the corresponding reference count (i.e., ground truth) in terms of mean absolute error (MAE) and mean percentage absolute error (MAPE).

### 3.4. Monte Carlo Propagation of Landmark-Localization Uncertainty

To assess the sensitivity of the proposed pipeline to uncertainty in landmark localization, a Monte Carlo uncertainty-propagation analysis [32,33] was carried out on 100 prediction instants randomly selected from the test dataset. For each selected instant, the corresponding causal input window was considered, namely the current frame together with the preceding frames required by the temporal model. The uncertainty assigned to the landmark coordinates was estimated from a static acquisition recorded while the subject maintained a fixed pose. Under these conditions, true facial motion can be assumed negligible; therefore, the variability observed in the estimated landmark coordinates can be interpreted as a measure of the repeatability of the landmark extraction process. For each landmark coordinate, the empirical standard deviation measured under static conditions was computed and adopted as the standard uncertainty associated with that input coordinate. For each selected causal input window, $N_{\mathrm{MC}}$ perturbed samples were generated by applying zero-mean Gaussian perturbations to all landmark coordinates in all frames of the window. Let $x_{l,c,\tau }$ denote coordinate c∈{x,y,z} of landmark *l* at frame τ. The perturbed coordinates for the *r*-th Monte Carlo sample were defined according to Equation (3), with $r=1,\ldots ,N_{\mathrm{MC}}$, where the perturbation term was sampled as in Equation (4). In this formulation, $\sigma_{l,c}^{\mathrm{static}}$ denotes the empirical standard deviation of landmark coordinate (l,c) estimated from the static acquisition.

(3) $${\tilde{x}}_{l,c,\tau }^{\left(r\right)}=x_{l,c,\tau }+\varepsilon_{l,c,\tau }^{\left(r\right)},$$

(4) $$\varepsilon_{l,c,\tau }^{\left(r\right)}∼N\;\left(0,\;{\left(\sigma_{l,c}^{\mathrm{static}}\right)}^{2}\right).$$

The perturbations were assumed independent across landmarks, coordinates, and frames. This assumption provides a simplified uncertainty model, as possible spatial and temporal correlations in landmark estimation errors were not explicitly modeled. The perturbations were applied before window-wise normalization, and each perturbed sample was propagated through the same preprocessing and inference pipeline used for the nominal input. For each perturbed sample, the model output probability associated with the considered prediction instant was recomputed. The resulting ensemble of output probabilities was then used to characterize the uncertainty propagated to the model output. For each prediction instant, the mean predicted probability, its standard deviation, and the empirical 95% interval were computed. These quantities were used to quantify the sensitivity of the predicted chewing probability to landmark-localization uncertainty under causal operating conditions.

### 3.5. Mobile App

To demonstrate deployment in a real-world scenario, the proposed pipeline was implemented as a native mobile application in Kotlin [34]. The application uses the front-facing camera to acquire facial video, detect the face, extract facial landmarks in real time, and estimate chewing activity directly on the device. The implementation is based on three main components: CameraX [35] for camera acquisition and preview, MediaPipe Face Mesh [20] for face-mesh extraction, and ONNX Runtime [31] for on-device execution of the trained recurrent model. For each frame, the detected facial landmarks are converted into a feature vector composed of the three-dimensional landmark coordinates, consistently with the representation adopted during model training (Section 3.2). The application maintains a temporal buffer matched to the selected model configuration. Once the buffer is filled, the same preprocessing and window-wise normalization procedure used during model development is applied, and the resulting tensor is forwarded to the ONNX model to estimate the chewing probability. Event counting in the mobile application follows the same post-processing logic described in Section 3.2. The user interface provides a live camera preview with optional facial landmark overlay, together with real-time feedback including the current chewing probability, cumulative chewing-event count, active inference backend, frame rate, and per-inference runtime. The mobile implementation used the same trained ONNX model adopted for the offline desktop experiments. No model simplification, pruning, retraining, or quantization was applied before deployment. The mobile application also retained the same landmark representation, temporal buffering, window-wise normalization, and event-level post-processing logic used in the offline pipeline. Therefore, the deployment test was intended to assess practical real-time execution of the trained pipeline on commercial Android hardware, rather than to introduce a separate mobile-specific model.

## 4. Results

This section reports the results of model selection, hold-out test evaluation, uncertainty analysis, and mobile deployment.

### 4.1. Model Training

As reported in Section 3.2, the adopted hardware completed 104 hyperparameter optimization trials over a period of 30 days. The final model was selected as the configuration yielding the lowest validation loss, evaluated in terms of weighted MSE. According to this criterion, trial no. 102 was identified as the final recurrent model, achieving a minimum weighted validation MSE of 0.043. The selected model was based on a GRU recurrent architecture. The complete hyperparameter configuration of the selected model is reported in Table 1. To examine the possible influence of subject-specific motion patterns, the selected configuration was also evaluated through an exploratory leave-one-subject-out validation, in which one subject was excluded in turn and the model was assessed across the resulting subject-wise folds. This analysis was performed using the same hyperparameters selected in trial no. 102. The resulting best validation losses obtained a mean weighted validation MSE of 0.046±0.001 across the five folds, compared with 0.043 for the selected model. The Bayesian optimization results also indicated that the selected architecture was not at the upper bound of the explored recurrent depth. Although the search space included models with up to eight recurrent layers, the best-performing configuration used four GRU layers, and no eight-layer architecture appeared among the ten best-performing trials. This suggests that increasing recurrent depth alone did not systematically improve validation performance within the explored search space.

**Figure 6:**
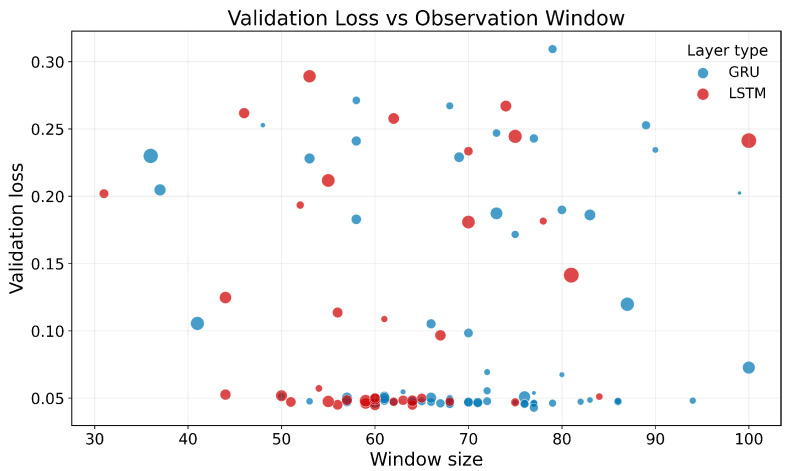
Validation loss as a function of the observation window size for the explored hyperparameter configurations. Each point corresponds to one optimization trial, and the marker colour identifies the recurrent layer type. The best-performing solutions are mainly concentrated in a window-size range from 50 to 80 samples, with the selected configuration corresponding to a GRU-based model with a window size of 77 samples (i.e., 2.56 s observation windows). Marker size is proportional to the number of optimization trials overlapping at the same window-size and validation-loss values, corresponding to models trained with different hyperparameter settings.

As shown in Figure 6, the best-performing models were consistently associated with observation windows ranging from 50 to 80 samples. This behavior is likely related to the characteristics of training videos acquired under real-world conditions, in which the subjects act naturally while speaking, chewing, and bringing objects such as food or utensils toward the mouth. These actions introduce frequent occlusions and temporal disturbances, making a broader temporal context beneficial for improving model performance.

As described in Section 3.2, the post-processing stage was calibrated by optimizing the decision threshold applied to the frame-wise predictions and the minimum duration required for a valid chewing event. The selected values were 0.78 for the threshold and 4 consecutive positive frames for the minimum event duration.

### 4.2. Model Performance Evaluation

The trained model was evaluated on an independent hold-out test set comprising five sessions and a total of 18,836 frames. Event-level performance was assessed by applying the same post-processing and event-matching protocol defined in Section 3.2, namely a probability threshold of 0.78 and a minimum event duration of 4 consecutive frames. Across the entire hold-out set, the proposed method detected 577 chewing events, compared with 589 ground truth events, thus showing a limited overall underestimation of the true chewing activity. For a direct comparison with the literature baseline, the implementation of the method proposed by Kim et al. [6] was applied to the same hold-out sessions. Table 2 reports the ground truth and predicted chew counts for each test session. Within the limited hold-out test set, the proposed method yielded counts close to the ground truth values in all sessions, whereas the Kim et al. method [6] showed a marked overestimation in most cases, particularly in Sessions 22 and 24, where an overestimation of 83 and 88 chewing events, respectively, occurred. The performance observed in Sessions 22 and 24 is likely related to the rule-based nature of the method by Kim et al. [6]. Since the approach relies on handcrafted peak-detection rules applied to a one-dimensional chewing-motion signal, it is inherently less robust to facial movements unrelated to chewing. During these two sessions, the subject was not only chewing but also speaking and performing other natural meal-related activities. Because such sources of variability are not explicitly modeled by the rule-based method, they can generate signal patterns that satisfy the decision rules and are therefore incorrectly counted as chewing events, leading to a marked overestimation of the chew count. A quantitative summary of the count-estimation performance is reported in Table 3. The proposed method achieved a MAE of 4.4 chews/session and a MAPE of 4.32%. By contrast, the literature baseline yielded a MAE = 39.4 and MAPE = 30.39%. In addition to count-based performance, the selected final model was evaluated using event-level detection metrics. Using the tolerance-based one-to-one matching criterion, the model yielded an event-level precision of 0.80, a recall of 0.79, and an F1 score of 0.80. These results indicate that the proposed method provided accurate aggregate chew counts and detected individual chewing events with a balanced trade-off between false detections and missed events.

As an additional robustness analysis, the models obtained from the exploratory leave-one-subject-out validation were evaluated on the same independent hold-out test set. Across the subject-wise models, the mean number of predicted chewing events was 561±28, compared with 589 sEMG-derived reference events. The corresponding event-level precision, recall, and F1 score were 0.81±0.01, 0.78±0.04, and 0.80±0.02, respectively. These results were consistent with those obtained by the selected final model and suggest relatively stable event-detection behavior across the available subject-wise training partitions. On the test workstation equipped with an Intel Core i9-14900KF CPU (Intel Corporation, Santa Clara, CA, USA) and an NVIDIA GeForce RTX 4090 GPU (NVIDIA Corporation, Santa Clara, CA, USA), the ONNX model was benchmarked on 1000 real windows sampled from hold-out test sessions. For a single real-time iteration, including window reshaping, NaN removal, per-window normalization, and batch-1 inference, the mean latency was 8.96 ± 5.74 ms on CPU and 6.89 ± 3.58 ms with ONNX Runtime CUDA execution.

### 4.3. Monte Carlo Uncertainty Analysis

**Figure 7:**
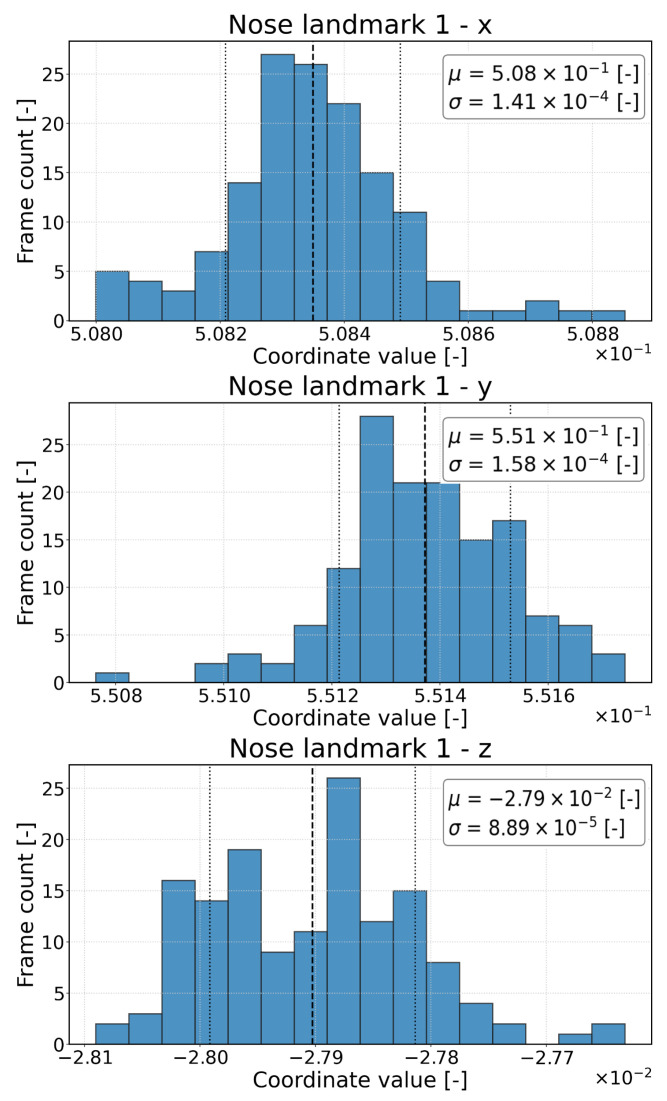
Coordinate distributions of the Nose landmark measured from the static acquisition used to estimate the input uncertainty.

**Figure 8:**
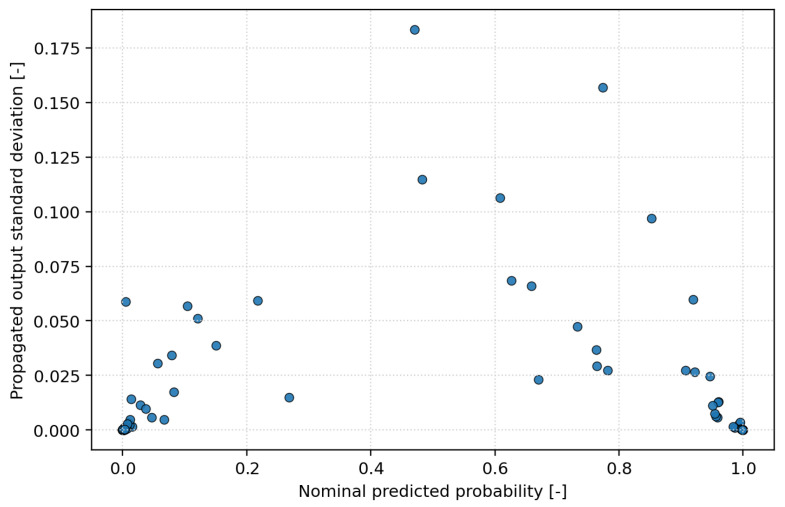
Propagated output standard deviation as a function of the nominal predicted chewing probability. The largest sensitivities are observed for intermediate probability values, whereas predictions close to 0 or 1 generally exhibit lower standard deviation.

**Figure 9:**
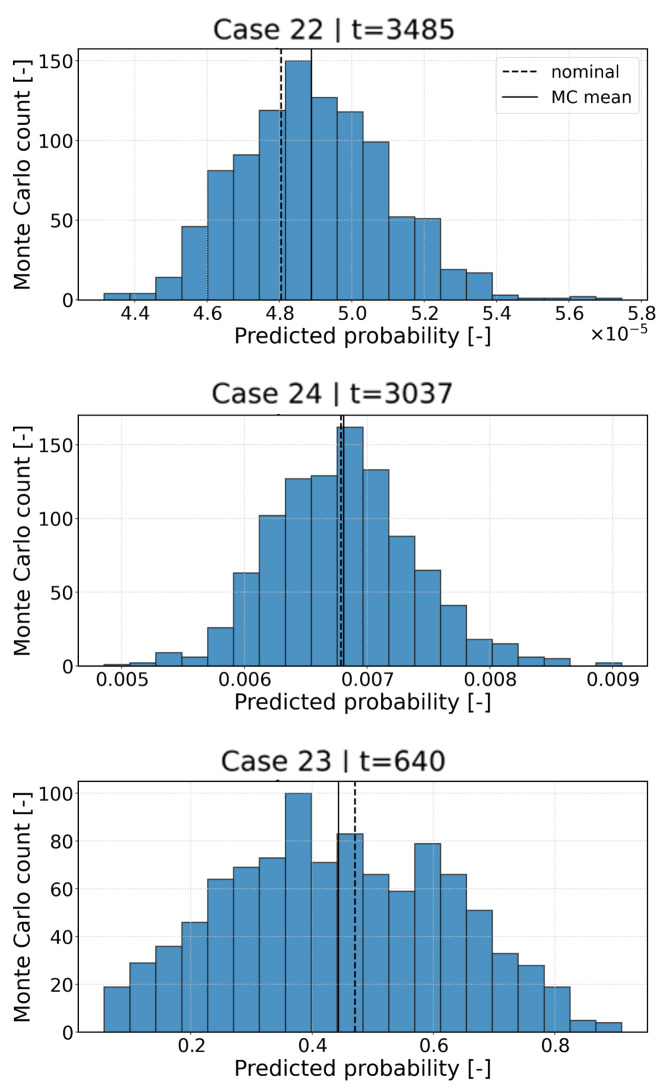
Examples of Monte Carlo output distributions for representative causal prediction instants with low, intermediate, and high propagated uncertainty. The corresponding output standard deviations are $\sigma_{y}=2.05\times 10^{-6}$, $5.77\times 10^{-4}$, and $1.83\times 10^{-1}$, respectively. In each panel, the dashed vertical line denotes the nominal prediction, while the solid vertical line denotes the Monte Carlo mean.

The Monte Carlo uncertainty propagation analysis was carried out as described in Section 3.4 on 100 causal prediction instants selected from the hold-out test sessions. For each instant, $N_{\mathrm{MC}}$ = 10,000 perturbed realizations were generated. Across all 468 landmarks, the empirical standard deviation of the input coordinates had mean values of $2.10\times 10^{-4}$ for *x*, $1.97\times 10^{-4}$ for *y*, and $9.99\times 10^{-5}$ for *z*. The corresponding median values were $1.56\times 10^{-4}$, $1.57\times 10^{-4}$, and $7.96\times 10^{-5}$, respectively. An example of the coordinate distributions obtained for the Nose landmark from the static acquisition used for input uncertainty estimation is shown in Figure 7. Across the 100 analyzed instants, the standard deviation of the predicted chewing probability had a mean value of $1.59\times 10^{-2}$ and a median value of $5.70\times 10^{-4}$. The discrepancy between mean and median indicates that, for most instants, the effect of landmark-localization uncertainty on the model output was limited, whereas a smaller number of instants exhibited appreciable sensitivity. The propagated output uncertainty was found to depend on the nominal predicted probability. As shown in Figure 8, instants associated with probabilities close to 0 or 1 generally exhibited lower standard deviation, while the largest propagated uncertainties were observed for intermediate probability values. The highest sensitivity was observed for case 23 at prediction index t=640, where the nominal predicted probability was 0.471, the propagated standard deviation was 0.183, and the empirical 95% interval was [0.108, 0.792]. Additional high-sensitivity instants were identified in cases 21 and 24. The five most sensitive instants are reported in Table 4. Representative examples of propagated output distributions are shown in Figure 9. Overall, uncertainty propagation remained limited for most of the analyzed instants, and the largest effects were localized mainly around intermediate probability values. From a practical standpoint, this result supports the use of a decision rule based not on isolated probability values, but on their temporal persistence above threshold. In this sense, the adopted event-level post-processing appears consistent with the observed uncertainty pattern, since it reduces the influence of locally unstable predictions and bases the final decision on temporally sustained chewing evidence.

### 4.4. Mobile App

**Figure 10:**
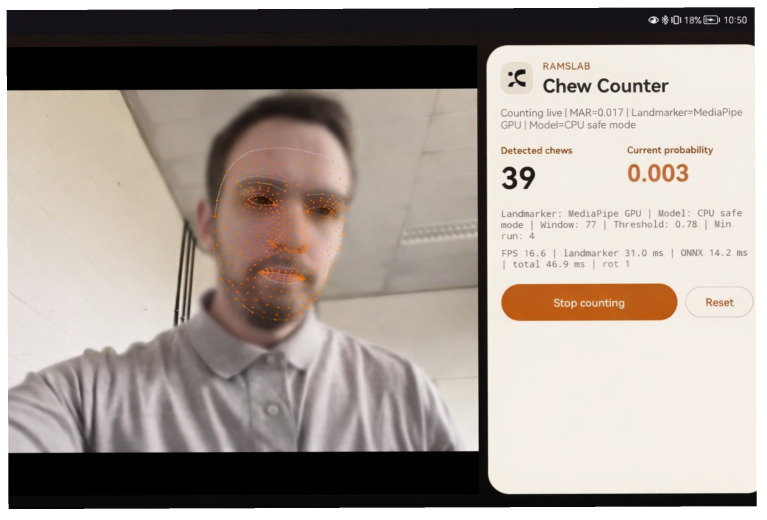
Representative screenshot of the developed mobile application during real-time chewing detection on the Huawei MatePad Pro tablet. The interface shows the live front-camera preview with facial landmark overlay, the cumulative number of detected chewing events, the current chewing probability, and the runtime measurements of the processing pipeline.

The selected model was successfully deployed within the developed Android application and tested on a Huawei MatePad Pro tablet (Huawei Technologies Co., Ltd., Shenzhen, China), equipped with a Kirin 990 chipset, 6 GB of RAM, and a Mali-G76 MP16 GPU. The application correctly performed frontal camera acquisition, real-time facial landmark extraction, online chewing-probability estimation, and cumulative chewing-event counting. The same event-detection rule adopted in the offline evaluation (Section 3.2) was retained in the mobile implementation, with a temporal window of 77 frames, a probability threshold of 0.78, and a minimum run length of 4 consecutive positive instants for event validation. Figure 10 reports a screenshot acquired during live operation on the main interface. The interface displays the camera preview, the facial landmark overlay, the cumulative number of detected chewing events, the current chewing probability, and the runtime information of the processing pipeline. During 15 min of continuous execution on the Huawei MatePad Pro tablet, the application sustained an average frame rate of 20 fps without interruptions. The average landmark extraction time was 32 ms, whereas the average ONNX inference time was 18 ms. Over the same interval, the battery level decreased from 68% to 66%, while the battery temperature increased from 24 °C to 26 °C. These observations suggest that the implementation can support short-term real-time operation on the tested device without evident battery- or thermal-related instability.

## 5. Conclusions

A causal video-based method for chewing-event detection was developed using facial landmark dynamics as input and sEMG-based labels as reference during dataset construction for model training. The experimental dataset included 108,013 frames from 21 acquisitions collected from different subjects in real meal conditions at 30 fps. Bayesian hyperparameter optimization was performed over 104 trials, and the selected architecture was a GRU with four recurrent layers, 164 hidden units, and a 77-frame temporal window. On the independent hold-out test set, comprising five sessions and 18,836 frames, the proposed method provided chewing-event counts in close agreement with the ground truth, with 577 detected events versus 589 reference events, a MAE of 4.4 chews/session, and a MAPE of 4.32%. At the event level, the final model achieved a precision of 0.80, recall of 0.79, and F1 score of 0.80 on the hold-out sessions, indicating that the agreement in aggregate chew counts was accompanied by temporally meaningful detection of individual chewing events. In comparison, the literature baseline by Kim et al. [6] showed substantially larger counting errors (MAE = 39.4, MAPE = 30.39%) and a marked tendency to overestimate chewing activity. These findings suggest that the proposed approach outperformed the rule-based baseline in the tested meal conditions, particularly when chewing co-occurs with speech and other natural facial movements. The uncertainty analysis quantified the stability of the full pipeline. Static-face repeatability produced average standard deviations of $2.10\times 10^{-4}$, $1.97\times 10^{-4}$, and $9.99\times 10^{-5}$ for the normalized *x*, *y*, and *z* coordinates, respectively. Propagation of these uncertainties through the model yielded a median standard deviation of the predicted chewing probability of $5.70\times 10^{-4}$ and a mean value of $1.59\times 10^{-2}$, with the largest effects observed close to intermediate probability values. The ONNX implementation showed low inference latency on the test workstation, with a mean processing time of 8.96 ± 5.74 ms on CPU and 6.89 ± 3.58 ms using ONNX Runtime with CUDA, supporting the feasibility of real-time deployment. Real-time operation was also preliminarily demonstrated on commercial hardware. The mobile Kotlin app implementation operated at 20 fps, with average execution times of 32 ms for landmark extraction and 18 ms for ONNX inference. From a privacy perspective, the final system does not require storage of the original video stream, as only the extracted facial landmarks are used for processing. The trained model should not be regarded as definitive, given the limited number of subjects, acquisitions, food types, and test sessions currently available. The reported performance, therefore, provides preliminary evidence that the proposed framework is feasible under the tested real-meal conditions, but it should not be interpreted as a population-level estimate of accuracy or as proof of robustness across all real-world eating scenarios. Moreover, the present study did not systematically quantify performance degradation as a function of head-pose angle or occlusion severity. Therefore, recordings involving substantial head rotations or strong and prolonged occlusions of the mouth or lower-face region should be considered outside the optimal operating conditions of the current framework. Similarly, robustness to specific illumination conditions, such as low light or backlighting, and to highly complex visual backgrounds was not quantitatively characterized. Overall, the proposed framework constitutes a promising basis for future developments, including dataset expansion, broader subject-independent validation, systematic assessment across food categories, controlled analysis of head-pose, occlusion, illumination, and background effects, and further refinement under more heterogeneous camera, device, and environmental conditions.

## Figures and Tables

**Table 1:** Final hyperparameter configuration of the selected recurrent model.

Parameter	Value
Recurrent layer type	GRU
Number of recurrent layers	4
Hidden units	164
Window size	77
Training stride	1
Dropout rate	0.150
Learning rate	$3.041\times 10^{-5}$
Batch size	40

**Table 2:** Reference sEMG and predicted chewing-event counts for each hold-out test session, comparing the proposed method and the literature baseline by Kim et al. [6].

Session	Ground Truth	Proposed Method	Kim et al. [6]
21	105	101	118
22	98	96	181
23	92	88	104
24	217	222	305
25	77	70	78
Total	589	577	786

**Table 3:** Overall chew-count estimation performance on the hold-out test sessions.

Method	MAE	MAPE (%)
Proposed method	4.4	4.32
Kim et al. [6]	39.4	30.39

**Table 4:** Most sensitive causal prediction instants identified by the Monte Carlo uncertainty analysis.

Session	Frame	Nom. Prob.	σ	95% CI
23	640	0.471	0.183	[0.108, 0.792]
21	1419	0.774	0.157	[0.373, 0.952]
23	1876	0.483	0.115	[0.280, 0.730]
21	2444	0.609	0.106	[0.363, 0.785]
24	3886	0.852	0.097	[0.595, 0.954]

## Data Availability

The original data presented in the study are openly available in Zenodo at https://doi.org/10.5281/zenodo.20112380, accessed on 24 May 2026.
